# Effects of foliar application of selenium and potassium-humate on oat growth in Baloza, North Sinai, Egypt

**DOI:** 10.1038/s41598-022-19229-x

**Published:** 2022-09-06

**Authors:** Rehab H. Hegab, Doaa Eissa, Ahmed Abou-Shady

**Affiliations:** 1 Soil Fertility and Microbiology Department, Water Resources and Desert Soils Division, Desert Research Center, El-Matariya, Cairo, 4540031 Egypt; 2 Soil Physics and Chemistry Department, Water Resources and Desert Soils Division, Desert Research Center, El-Matariya, Cairo, 4540031 Egypt

**Keywords:** Environmental impact, Ecosystem ecology

## Abstract

In this study, the effects of foliar application of selenium (Se) at different concentrations were examined based on changes in several parameters such as nitrogen, phosphorous, and potassium (NPK) concentration in soil and oat plant, oat yield, organic matter in the soil (OMS), non-enzymatic antioxidants, and total phenol content. Chromium (Cr), iron (Fe), manganese (Mn), zinc (Zn), and copper (Cu) concentrations were also assessed in oat straw and seeds. The study complies with local and national guideline. Simultaneous application of potassium humate (K-humate) with Se was also investigated in this study. Se application increased the bioavailability of N and P in soil and their total concentration in the straw and seeds of each plant. Se concentrations were proportional to the amount of phosphorous found in soil (P-soil) but not with K concentrations in seed (K-plant). Application of K-humate with Se increased the bioavailable fraction of K-soil; however, it did not increase the bioavailable fraction of K-straw or K-seed. Although the application of Se alone substantially enhanced yield, the simultaneous application of K-humate showed no additional effect. Moreover, responses of seed yield and plant length were not significant after the application of Se with or without K-humate. OMS and total phenol content were proportional to the application rate of Se with and without K-humate. Non-enzymatic antioxidant content was also proportional to Se concentrations but not proportional to K-humate. The total Se concentrations in the soil, plant straw, and seeds increased with the addition of K-humate. Furthermore, the total Cr concentrations were reduced after the application of Se and K-humate. Fe concentration in the straw and seeds varied from one treatment to another, and Mn concentration was reduced in response to the foliar application of Se and K-humate. Zn concentrations in the straw and seeds of plants were reduced with the application of varying concentrations of Se. Increasing the application rate of Se induced a reduction in the Cu concentration in seeds. In contrast, the simultaneous application of Se and K-humate increased the Cu concentration in seeds.

## Introduction

Research on selenium (Se) began when Schwartz and Foltz found that Se in fodder prevented liver cirrhosis and muscular dystrophy in rats^1^. Based on its antioxidant and anticancer properties, Se has various functions such as acting as an antioxidant in plants^2^.

Plant growth does not depend on the Se concentration available in soil. However, Se concentrations in human food and animal feed have important health implications^3^. The boundary between Se concentrations that fulfill essential nutritional requirements and toxic Se concentrations is narrow and is affected by the chemical form and environmental conditions^2^. Se may modify the ability of plants to tolerate UV-induced oxidative stress, promote the growth of aging seedlings, and delay senescence. Se nanoparticles (SeNPs) affected the growth of groundnut cultivars by altering photosynthetic pigments, total soluble sugars, antioxidant enzymes (ascorbic acid peroxidase, catalase, and peroxidase), phenol content, total flavonoids, and lipid peroxidation. In contrast, sandy soil conditions enhanced plant tolerance after the application of SeNPs as a stressor or a stimulant^4^. Se application also reversed the negative salinity effect on photochemical efficiency^2^. Se additive application reduced the occurrence of adverse responses caused by heavy metals, heat, ultraviolet(UV)-B, cold, salt stress, and drought^5^.

Organic fertilizers, such as potassium humate (KHM) and potassium fulvic acid (BSFA) are used to prevent plant diseases, improve soil structure, and augment soil nutrient levels^6^. KHM and BSFA addition reshaped microbial functions and nutrient levels were found to increase in ginseng soil^6^. Furthermore, the application of KHM enhanced seed germination, nutrient uptake, and the growth of seedlings^7^.

The oat plant (*Avena sativa L.*) is rich in a variety of antioxidant compounds, such as avenanthramides, vitamin E (tocols), phenolic compounds, and phytic acid. Moreover, flavonoids and sterols are primarily found in the outer layers of the oat kernel^8^. Commercial plant-based dairy alternatives are manufactured using oats such as beverages and yogurt-like products. However, a drawback of these products is their low protein content, ~ 0%–1%, when oat is the main source^9^. Starch derived from oats has attracted attention for its potential use in various food and nonfood applications^10^. A serious environmental pollutant may be caused by Cr that may be derived through its wide industrial use that becomes of recent concern. Plants toxicity caused by Cr relies on its valence state (e.g., Cr(III) is less toxic, however, Cr(VI) is highly toxic and mobile). Owing to the lack of a specific transport system of plants for Cr, it is subject to be taken up by carriers of essential ions (e.g., iron or sulfate). The harmful effect of Cr on plant growth and development is found in the alterations germination process, in addition to, roots, stems, and leaves growth that ultimately affects the amounts of dry matter production and yield. Also, deleterious effects may be caused by Cr (e.g., water relations, photosynthesis, and mineral nutrition), as well as, metabolic alterations through its effect on enzymes or other metabolites, as well as, its oxidative stress^11^.

This study assessed different foliar applications of Se (12 × 10^−3^, 63 × 10^−3^, and 88 × 10^−3^ mM) on the productivity of oat plants. Study endpoints included the nitrogen, phosphorous, and potassium (NPK) concentration in soil and oat plants, oat yield, organic matter in soil (OMS), antioxidant and total phenols, Se concentrations in soil and oats, and chromium (Cr), iron (Fe), manganese (Mn), zinc (Zn), and copper (Cu) concentrations in oat straw and seeds. Simultaneous application of Se with K-humate was also investigated in parallel.

## Materials and methods

### Soil preparation

In the North Sinai area of Baloza, Egypt, soil samples were collected from two depth intervals of 0–30 cm and 30–60 cm. Plant samples were taken from a private land and permission was obtained for sampling of plants as well as the study complies with local and national guidelines. Soils were air-dried, crushed, and sieved through a 2 mm mesh. The international pipette method was used to assess soil texture. Soil organic matter content was measured as previously described^12^. Some chemical and physical properties of the studied soils are listed in Table 1. pH and electrical conductivity were measured in the soil paste, and element content was analyzed using inductively coupled plasma-optical emission spectroscopy (ICP) after digestion with a mixture of HNO_3_, H_2_SO_4_, and HClO_4_ as previously described^13^. Total non-enzymatic antioxidant and total phenol levels were measured as previously described^14^.

**Table 1:** Some chemical and physical properties of the studied soils.

Depth	0–30 cm	30–60 cm
**Particle size distribution (%)**		
Sand	85.10	88.70
Silt	8.35	6.57
Clay	6.55	4.73
Texture class	Sandy	Sandy
pH saturated soil paste	8.13	8.02
EC(ds m^−1^)	1.39	1.05
**Soluble ions in saturated soil extract (meq L**^**−1**^**)**		
Na	5.11	2.69
K	0.44	3.17
Ca	3.85	4.13
Mg	4.50	0.58
Cl	3.39	3.72
HCO_3_	3.55	3.37
SO_4_	6.96	3.41
**Available nutrients (mg kg**^**−1**^**)**		
N	12.60	19.12
P	4.56	1.84
K	54.3	34.1
**Some total trace elements (mg kg**^**−1**^**)**		
Se	0.24	0.20
Cr	70.4	66.3
Fe	12,746	14,398
Mn	181.6	174.0
Zn	102.4	116.9
Cu	21.6	32.6
OM (%)	0.12	0.11

### Field experiment

Field experiments were performed during cropping season 2020–2021 to understand the effect of Se and K-humate application on non-enzymatic the antioxidant content and yield of oats (*Avena sativa*) in Baloza, North Sinai, Egypt. Fertilizers were applied at constant rates in all experiments. Ammonium sulfate, calcium superphosphate, potassium sulfate, and biofertilizers were used. Experiments were based on a split-plot design with three replicates and foliar application of Se at concentrations of 12 × 10^−3^, 63 × 10^−3^, and 88 × 10^−3^ mM with and without K-humate. The source of Se was sodium selenite (Na_2_SeO_3_). The source of K-humate was potassium humate (C_9_H_8_K_2_O_4_—2.3 mM). Plants were cut at the soil surface 120 days after planting and washed with deionized water. Following this, the plants were oven-dried at 70 °C for 48 h, weighed for their dry matter yield, and then ground. Plants and soil samples obtained after the application of different treatments were digested as previously described^13^ and analyzed using ICP. NPK was also analyzed using these digestions^15^. Available N in soil samples was extracted by adding 2 M potassium chloride as previously described^16^. The available K and P were extracted with DTPA and ammonium bicarbonate as previously described^17^.

### Statistical analysis

Data were statistically analyzed, and means were compared using the least significant differences. Results were considered to be statistically significant at *p* < 0.05 (Statistic version 9). Statistic version 9 was used for analyses and customizable graphs were generated. Details of the program are available online^18^.

## Results and discussion

### Effects of Se and K-humate on nitrogen concentrations

The N concentration in the soil varied in availability and total content in oat straw and seeds after the foliar application of Se and K-humate. Se alone increased the availability of N in the soil in the following order: Se3 > Se2 > Se1 > control. Thus, Se was found to increase the available N-soil in an application-rate-dependent manner (Table 2). The availability of N-soil after Se application was improved via the simultaneous application of K-humate with the same rate-dependence as observed with Se alone. Comparable results were found using the sum of means for analysis. The insignificant difference found between the sum of means for control and treatment at an Se concentration of 12 × 10^−3^ mM Se may reflect the relatively low concentration of Se used.

**Table 2:** Effect of selenium and K-humate on nitrogen content.

Treatments	N-soil (mg kg^−1^)			N-straw (mg kg^−1^)			N-seeds (mg kg^−1^)		
	NH*	H**	Mean	NH	H	Mean	NH	H	Mean
C	19.13E	22.52DE	20.82C	0.24C	0.34C	0.29B	1.53D	1.96CD	1.74C
Se1	25.90CDE	30.17CDE	28.03C	0.43BC	0.43BC	0.43B	2.05BC	2.08BC	2.06B
Se2	33.36CD	54.14B	43.77B	0.65BC	1.32A	0.99A	2.28ABC	2.41AB	2.34A
Se3	37.03C	69.52A	53.27A	0.86AB	1.32A	1.09A	2.37AB	2.57A	2.47A
Mean	28.85B	44.09A		0.54B	0.85A		2.06A	2.25A	
LSD at 5%	T1 = 9.16	T2 = 4.74	T1 × T2 = 11.35	T1 = 0.27	T2 = 0.29	T1 × T2 = 0.49	T1 = 0.15	T2 = 0.25	T1 × T2 = 0.38

*Refers to Se application without K-humate.

**Refers to the simultaneous application of Se and K-humate.

The total N-straw content increased as a result of an increased content of N-plant (Table 2). Differences were found to be insignificant between Se concentrations of 12 × 10^−3^ mM, 63 × 10^−3^ mM, and controls. Likewise, the simultaneous application of K-humate showed insignificant differences between Se concentrations of 63 × 10^−3^ mM and 88 × 10^−3^ mM. Insignificant differences were noted between the control and Se concentration of 12 × 10^−3^ mM and the Se concentration of 63 × 10^−3^ and 88 × 10^−3^ mM using the sum of means. The total N-seeds content increased for application rates of 12 × 10^−3^–88 × 10^−3^ mM, and the simultaneous application of K-humate augmented this increase. The application rate dependency of the effects of Se and K-humate application was identical to that observed in N-soil and N-straw. No significant differences among Se and K-humate applications were observed. An insignificant difference was observed among the sum of means for Se and K-humate applications at concentrations of 63 × 10^−3^ and 88 × 10^−3^ mM.

The application of Se caused proportional increases in N-soil, N-straw, and N-seeds, and the simultaneous application of K-humate improved this effect. Previously, the application of Se resulted in an increase in the accumulation of NPK which altered N and K distribution. However, the distribution of P was not affected^19^. Furthermore, the application of Se ultimately resulted in an increase in the accumulation of N, calcium (Ca), K, and Mn^20^. A significant increase in concentrations of N and S in the rice grain plants grown under N-limiting conditions was also observed while the Ca that have been treated with Se regardless of N supply^21^. Thus, a synergistic interaction between Se and N in total grain proteins was reported^21^.

### Effects of Se and K-humate on P

The effect of applications of different Se concentrations without K-humate on the available P-soil showed a reduction in the following order: Se3 > Se2 > Se1 > control (Table 3). Thus, the foliar application rate of Se caused a rate-dependent increase in the available P-soil. Simultaneous application of K-humate further increased P-soil availability. A rate dependency similar to Se alone was also observed with simultaneous Se and K-humate application. A similar result was observed using the sum of means for data analysis. Significant differences were observed among all treatments.

**Table 3:** Effect of selenium and K-humate on phosphorous content.

Treatments	P-soil (mg kg^−1^)			P-straw (mg kg^−1^)			P-seeds (mg kg^−1^)		
	NH*	H**	Mean	NH	H	Mean	NH	H	Mean
C	7.60D	9.58D	8.59D	0.08C	0.10BC	0.09B	1.23D	1.68C	1.45C
Se1	13.01C	14.78C	13.89C	0.12ABC	0.12ABC	0.12AB	1.76BC	1.77BC	1.77B
Se2	15.72C	22.70B	19.21B	0.13ABC	0.14ABC	0.13AB	1.80BC	2.00AB	1.90AB
Se3	21.32B	33.07A	27.19A	0.15AB	0.16A	0.16A	1.92ABC	2.11A	2.02A
Mean	14.41B	20.03A		0.12A	0.13A		1.68B	1.89A	
LSD at 5%	T1 = 2.80	T2 = 1.17	T1 × T2 = 3.25	T1 = 0.04	T2 = 0.02	T1 × T2 = 0.05	T1 = 0.19	T2 = 0.14	T1 × T2 = 0.28

*Refers to Se application without K-humate.

**Refers to the simultaneous application of Se and K-humate.

Foliar application of Se increased total P-straw. An insignificant difference was found between the control and Se concentrations of 12 × 10^−3^ and 63 × 10^−3^ mM, which was similar to findings observed after the application of K-humate. Moreover, insignificant differences were observed between the applications of Se and Se + K-humate. An insignificant effect was found between control and Se concentrations of (12 × 10^−3^ and 63 × 10^−3^ mM), and K-humate application using the sum of means.

The application of Se having concentrations ranging from 12 × 10^−3^ to 88 × 10^−3^ mM resulted in increased P-seeds and the addition of K-humate augmented this effect (Table 3). The effect of Se and K-humate applications showed a decrease in the following order: Se3 > Se2 > Se1 > control. Insignificant differences between values were observed when Se was applied without K-humate at concentrations of 12 × 10^−3^ and 63 × 10^−3^ mM, and for the sum of means for Se and K-humate applications at concentrations of 12 × 10^−3^ and 63 × 10^−3^ mM. Thus, the application rate of Se caused a proportional increase in P-soil, P-straw, and P-seeds. Furthermore, the simultaneous application of K-humate augmented this effect.

Consistently, concentrations of P and Ca increased in response to the application of selenite-Se (Na_2_SeO_3_⋅5H_2_O) to maize seedlings^22^, and the application of Se led to an increase in the accumulation of NPK, with alteration of N and K distribution. However, the distribution of P was not influenced^19^.

### Effects of the foliar application of Se and K-humate on K

Different application rates of Se without humate increased K-soil and this effect showed a decrease in the following order: Se3 > Se2 > Se1 = control (Table 4). Again, the foliar application rate of Se causes a proportional increase, in this case, in K-soil. The application of K-humate with Se augmented this effect. A similar rate dependency was also observed with simultaneous application and when the sum of means was used. An insignificant difference was observed between the sum of means for controls and Se concentrations of 12 × 10^−3^ mM.

**Table 4:** Effect of selenium and K-humate on potassium content.

Treatments	K-soil (mg kg^−1^)			K-straw (mg kg^−1^)			K-seeds (mg kg^−1^)		
	NH*	H**	Mean	NH	H	Mean	NH	H	Mean
C	72.31D	74.82D	73.56C	0.69A	0.75A	0.72A	0.34A	0.41A	0.37A
Se1	78.88D	85.65CD	82.27C	0.83A	0.90A	0.87A	0.42A	0.42A	0.42A
Se2	98.25BC	108.98B	103.62B	0.90A	1.01A	0.95A	0.46A	0.49A	0.47A
Se3	101.33B	132.75A	117.04A	1.01A	1.07A	1.04A	0.48A	0.49A	0.48A
Mean	87.69B	100.55A		0.86A	0.93A		0.42A	0.45A	
LSD at 5%	T1 = 12.14	T2 = 5.99	T1 × T2 = 14.80	T1 = 0.42	T2 = 0.37	T1 × T2 = 0.68	T1 = 0.12	T2 = 0.08	T1 × T2 = 0.17

*Refers to Se application without K-humate.

**Refers to the simultaneous application of Se and K-humate.

The foliar application of Se led to a slight increase in the total K-straw content (Table 4). An insignificant change was observed for Se concentrations from 12 × 10^−3^ to 88 × 10^−3^ mM, and similar results were found with the additional application of K-humate.

The application of Se at concentrations from 12 × 10^−3^ to 88 × 10^−3^ mM resulted in a slight increase in K-seeds, and the additional application of K-humate only slightly increased the accumulation of K (Table 4). An insignificant difference was observed between Se alone and with K-humate. Similar findings were noted when the sum of means was used for analysis. Se application rates thus produce a proportional increase in K-soil but not in K-straw or K-seeds. Comparable data were noted after K-humate addition. Concentrations of K previously decreased in response to selenite-Se (Na_2_SeO_3_⋅5H_2_O) application to maize seedlings; however, magnesium (Mg) concentrations did not change^22^. Moreover, the application of Se led to the accumulation of NPK and altered N and K distribution without affecting the P distribution^19^. Consistently, the application of Se ultimately resulted in increasing K accumulation^20^.

### Effects of Se and K-humate application on oat growth

Application of Se improved the yield, which was assessed as kg × 10^−3^/feddan (Table 5). Higher concentrations of Se produced a higher yield of oat. The effect of Se showed a reduction in the following order: Se3 > Se2 > Se1 > control. The simultaneous application of K-humate increased the yield only slightly, resulting in insignificant differences. Similar findings were also observed when the sum of means was used. In contrast, seed production was not significantly affected, and plant length (m × 10^–2^) did not show a significant response. In contrast, Se application to potato plants enhanced tuber yield, plant growth, and quality compared with controls. Moreover, Se application along with different N additions ultimately increased potato productivity compared with Se or N alone^23^. Similarly, the grain yield increased when Se was applied; this application was significant at low levels^24^.

**Table 5:** Effect of Se and K-humate application on oat growth.

Treatments	Yield/fed (kg × 10^–3^/feddan)			Seeds/fed (kg × 10^–3^/feddan)			Length (m × 10^–2^)		
	NH*	H**	Mean	NH	H	Mean	NH	H	Mean
C	5.09C	5.15C	5.12C	1.79A	2.22A	2.00A	101.0A	102.0A	101.5A
Se1	5.82BC	7.95ABC	6.88BC	2.25A	2.35A	2.30A	102.0A	103.0A	102.5A
Se2	8.66ABC	8.79AB	8.72AB	2.39A	2.49A	2.44A	104.0A	104.3A	104.1A
Se3	9.52A	10.93A	10.22A	2.57A	2.80A	2.68A	104.3A	105.0A	104.6A
Mean	7.27A	8.20A		2.25A	2.46A		102.83A	103.5A	
LSD at 5%	T1 = 3.13	T2 = 1.21	T1 × T2 = 3.57	T1 = 0.87	T2 = 0.38	T1 × T2 = 1.03	T1 = 3.52	T2 = 2.34	T1 × T2 = 4.83

*Refers to Se application without K-humate.

**Refers to the simultaneous application of Se and K-humate.

### Effects of Se and K-humate applications on OMS (%) and non-enzymatic antioxidants and total phenols in oat plants

The total OMS content increased with increasing Se concentrations, perhaps due to stimulation of root growth or microbial biomass. This effect showed a decrease in the following order: Se3 > Se2 > Se1 > control. The addition of K-humate by foliar application significantly augmented the OMS content (%) (Table 6). Application of Se also increased the non-enzymatic antioxidant content; however, the increases were insignificant at Se concentrations of 12 × 10^−3^ and 63 × 10^−3^ mM. The highest values for non-enzymatic antioxidants were observed at Se concentrations of 88 × 10^−3^ mM. The application of K-humate along with Se did not significantly augment the effects observed after the application of Se alone. Analyses using the sum of means were completely consistent with these findings.

**Table 6:** Effect of selenium and K-humate application on organic matter in soil (OMS), non-enzymatic antioxidant, and total phenols in oats.

Treatments	OMS (%)			Non-enzymatic antioxidant (μg AAE /mg ext.)			Total phenols (μg GAE/mg ext.)		
	NH*	H**	Mean	NH	H	Mean	NH	H	Mean
C	0.11H	0.16G	0.14D	91.20C	97.2BC	94.20C	2.46D	2.93CD	2.72C
Se1	0.22F	0.28E	0.25C	115.7ABC	135.2ABC	125.45B	3.07CD	3.15C	3.11BC
Se2	0.33D	0.44B	0.38B	141.7ABC	148.7AB	145.20AB	3.22C	3.59BC	3.40B
Se3	0.39C	0.56A	0.47A	150.7A	154.2A	152.45A	3.94B	4.89A	4.39A
Mean	0.26B	0.36A		124.8A	133.8A		3.17B	3.64A	
LSD at 5%	T1 = 0.04	T2 = 0.009	T1 × T2 = 0.04	T1 = 21.44	T2 = 34.08	T1 × T2 = 52.74	T1 = 0.40	T2 = 0.38	T1 × T2 = 0.68

*Refers to Se application without K-humate.

**Refers to the simultaneous application of Se and K-humate.

Se positively enhanced the total phenol content with effects decreasing in the following order: Se3 > Se2 > Se1 > control. Furthermore, this effect was significantly amplified with the simultaneous application of K-humate. Analysis using the sum of means gave comparable results. Se enhances the ability of plants to cope with stress by stimulating plant cell antioxidant capacity though the upregulating of antioxidant enzymes, such as CAT, SOD, and GSH-Px. Se also increases the synthesis of PCs, GSH, proline, ascorbate, alkaloids, flavonoids, and carotenoids. Se may also induce the spontaneous dismutation of the superoxide radical into H_2_O_2_. Elevated antioxidant capacity can reduce lipid peroxidation by lowering ROS accumulation under metal-induced oxidative stress conditions^25^. Application of Se using foliar spray also induced an increase in the concentration of rosmarinic acid^20^.

### Effects of Se and K-humate applications on Se content

After the application of Se, Se-soil concentrations increased. The effects of Se concentrations decreased in the following order: Se3 > Se2 > Se1 > control. The additional application of K-humate significantly amplified these effects (Table 7). The treatment of K-humate that increased Se content in the soil may be owing to experimental errors, however, increasing Se content in either straw or seeds may be owing to the increased stimulating movement from soil to different parts of the plant. Se-straw content increased with increasing the Se foliar application; this effect decreased in the following order: Se3 > Se2 > Se1 > control. The simultaneous application of K-humate augmented the effects observed after the application of Se alone. Total Se concentration also increased Se-seeds like Se-straw for Se alone, Se with K-humate, and using the sum of means for analysis.

**Table 7:** Effects of Se and K-humate applications on Se content.

Treatments	Se-soil (mg kg^−1^)			Se-straw (mg kg^−1^)			Se-seeds (mg kg^−1^)		
	NH*	H**	Mean	NH	H	Mean	NH	H	Mean
C	0.28G	1.03F	0.66D	0.06G	1.11F	0.59D	0.23G	0.97F	0.60D
Se1	1.12EF	1.60E	1.36C	1.27EF	1.71DE	1.49C	1.52E	1.82D	1.67C
Se2	3.46D	4.33C	3.90B	2.02D	2.57C	2.30B	2.27C	2.57B	2.42B
Se3	5.32B	6.24A	5.78A	3.96B	4.77A	4.36A	2.77B	3.30A	3.04A
Mean	2.55B	3.30A		1.83B	2.54A		1.70B	2.17A	
LSD at 5%	T1 = 0.36	T2 = 0.30	T1 × T2 = 0.56	T1 = 0.40	T2 = 0.25	T1 × T2 = 0.54	T1 = 0.28	T2 = 0.12	T1 × T2 = 0.33

*Refers to Se application without K-humate.

**Refers to the simultaneous application of Se and K-humate.

### Effects of Se and K-humate application on Cr content

The highest concentrations of Cr were observed in control plants followed by Se2 > Se3 > Se1. In response to Se application, the Cr-straw content decreased (Table 8). The difference between Se2 and Se3 was insignificant. K-humate addition induced a notable increase in Cr-straw in the following order: control > Se3 > Se2 > Se1. This may be owing to the increased stimulating movement of Cr from soil to different parts of the plant. Results obtained from Se treatments varied depending on the presence of K-humate. Cr-seeds decreased in the following order: Se2 > Se3 > Se2 > control. The addition of K-humate increased the Cr-seed content compared with Se alone; however, the difference between Se2 and Se3 was insignificant. Analysis using the sum of means did not produce significant differences.

**Table 8:** Effects of Se and K-humate application on Cr content.

Treatments	Cr-straw (mg kg^−1^)			Cr-seeds (mg kg^−1^)		
	NH*	H**	Mean	NH	H	Mean
C	38.58ABC	51.67A	45.12A	37.56B	51.44A	44.50A
Se1	27.38C	29.57C	28.47C	43.86AB	47.44AB	45.65A
Se2	37.35BC	32.57BC	34.96BC	46.01AB	53.62A	49.82A
Se3	36.77BC	46.16AB	41.47AB	46.48 AB	54.49A	50.48A
Mean	35.02A	39.99A		43.48 B	51.75A	
LSD at 5%	T1 = 9.04	T2 = 7.53	T1 × T2 = 13.96	T1 = 7.93	T2 = 5.67	T1 × T2 = 11.27

*Refers to Se application without K-humate.

**Refers to the simultaneous application of Se and K-humate.

### Effects of Se and K-humate applications on Fe content

Variable effects were produced using different application rates of Se on Fe-straw, and this effect was observed in the following order: Se3 > Se1 > control > Se2 (Table 9). Differences were insignificant among control, Se1, and Se2. K-humate caused concentrations of Fe-straw to significantly increase in the following order: control > Se3 > Se2 > Se1. Differences between control and Se3 as well as Se1 and Se2 were insignificant. Analysis using the sum of means was similar. Neither Se nor Se with K-humate applications produced significant changes in Fe-seeds. Analysis using the sum of means was similar. Low concentration of Se application may enhance plant productivity and encourage phytoremediation by improving plant tolerance to stress and enhancing photosynthesis^25^. Further, a significant increase was observed in concentrations of Fe and S in rice grain grown in N-limiting conditions while Ca that have been treated with Se regardless of N supply^21^.

**Table 9:** Effects of Se and K-humate applications on Fe content.

Treatments	Fe-straw			Fe-seeds		
	NH*	H**	Mean	NH	H	Mean
C	3603B	6292A	4948A	8552A	9076A	8814A
Se1	3332B	3544B	3438B	9171A	9580A	9375A
Se2	2920B	3922B	3421B	9342A	8908A	9125A
Se3	4737AB	5821A	5279A	9365A	9156A	9260A
Mean	3648B	4895A		9108A	9180A	
LSD at 5%	T1 = 1498	T2 = 784.2	T1 × T2 = 1863	T1 = 650.8	T2 = 794.3	T1 × T2 = 1297.8

*Refers to Se application without K-humate.

**Refers to the simultaneous application of Se and K-humate.

### Effects of Se and K-humate application on Mn content

Application of Se reduced the Mn-straw content, and this effect was observed in the following order: control > Se2 > Se1 > Se3. No significant difference was found between control and Se1 (Table 10). In contrast, K-humate addition further reduced Mn-straw concentrations in the following order: control > Se1 > Se3 > Se2. The control and Se1 were not significantly different when using the sum of means for analysis. Likewise, no significant difference was seen between Se1 and Se3. Accumulation of Mn in seeds varied among treatments in the following order: control > Se2 > Se3 > Se1. K-humate addition altered this order to be in the following order: control > Se2 > Se1 > Se3. No significant differences were observed between Se2 and Se3 when the sum of means for analysis was used. Previously, the application of Se increased the concentrations of Mg and molybdenum in grains grown in 16 and 24 mM N compared with N-limited plants^21^.

**Table 10:** Effects of Se and K-humate application on Mn content.

Treatments	Mn-straw (mg kg^−1^)			Mn-seeds (mg kg^−1^)		
	NH*	H**	Mean	NH	H	Mean
C	100.26AB	89.10AB	94.67A	154.66A	155.29A	154.98A
Se1	82.10AB	85.80AB	83.95AB	139.93B	149.79AB	144.86B
Se2	110.28A	28.00C	69.14B	153.87A	151.08AB	152.48AB
Se3	74.77B	82.42AB	78.59AB	145.36AB	148.53AB	146.95AB
Mean	91.85A	71.32B		148.45A	151.17A	
LSD at 5%	T1 = 25.00	T2 = 13.76	T1 × T2 = 31.67	T1 = 8.74	T2 = 7.24	T1 × T2 = 13.47

*Refers to Se application without K-humate.

**Refers to the simultaneous application of Se and K-humate.

### Effect of Se and K-humate applications on Zn content in oat plants

Application of Se2—the middle concentration of Se—resulted in highest accumulation in Zn-straw, and this effect was observed in the following order: Se2 > Se1 > control > Se3 (Table 11). The application of K-humate with Se resulted in some insignificant variations compared with the application of Se alone. Control, Se1, and Se3 were insignificantly different when the sum of means was used for the analysis. Concentrations of Zn in seeds were reduced after Se application. K-humate with Se foliar application altered the concentration of Zn in seeds with impacts in the following order: control > Se3 > Se1 > Se2. The difference between Se1 and Se3 was insignificant. Additionally, insignificant differences in Zn concentrations after application of Se1, Se2, and Se3 were found when the sum of means was used for analysis. Low concentrations of Se possibly enhance plant productivity and phytoremediation capacity by improving the ability of plants to tolerate stress and enhancing photosynthesis^25^.

**Table 11:** Effect of Se and K-humate applications on Zn containing oat plant.

Treatments	Zn-straw (mg kg^−1^)			Zn-seeds (mg kg^−1^)		
	NH*	H**	Mean	NH	H	Mean
C	16.43BC	18.35AB	17.39AB	78.65A	68.05AB	73.35A
Se1	21.01AB	9.54C	15.27B	58.35ABC	47.82BC	53.08B
Se2	28.56A	13.77BC	21.17A	64.24ABC	37.11C	50.67B
Se3	15.42BC	20.67AB	18.05AB	65.32ABC	53.78ABC	59.55AB
Mean	20.36A	15.58A		66.64A	51.69A	
LSD at 5%	T1 = 4.96	T2 = 5.18	T1 × T2 = 8.85	T1 = 18.96	T2 = 17.17	T1 × T2 = 30.80

*Refers to Se application without K-humate.

**Refers to the simultaneous application of Se and K-humate.

### Effects of Se and K-humate application on Cu content

Increasing concentrations of Se from 12 × 10^−3^ to 88 × 10^−3^ mM increased the concentration of Cu-seed, and this effect was observed in the following order: Se1 > control > Se2 > Se3 as it shown in Table 12. Application of Se with K-humate showed significant changes in the Cu-straw content in the following order: Se1 > Se2 > control > Se3. No significant differences were observed using the sum of means for analyses. In contrast, the foliar application of Se resulted in increases in Cu-seed at concentrations of Se1 and Se3; however, at 63 × 10^−3^ mM (Se2), a reduction in Cu-seed was observed. K-humate with Se simultaneously resulted in increased Cu-seed content with impacts decreasing in the following order: Se3 > Se1 > control > Se2. The sum of means analysis showed no significant variation between control and Se2. Previously, the application of Se led to a decrease in the concentrations of Cu in grains grown in 16 and 24 mm N compared with N-limited plants^21^.

**Table 12:** Effects of Se and K-humate application on Cu content.

Treatments	Cu-straw (mg kg^−1^)			Cu-seeds (mg kg^−1^)		
	NH*	H**	Mean	NH	H	Mean
C	11.99AB	10.65AB	11.32A	6.49F	14.21CDE	10.35C
Se1	14.39A	12.00AB	13.19A	17.33BCD	21.33BC	19.33B
Se2	10.60AB	11.66AB	11.13A	6.75EF	12.63DEF	9.69C
Se3	7.25B	7.71AB	7.48A	24.33AB	29.47A	26.90A
Mean	11.06A	10.51A		13.72B	19.41A	
LSD at 5%	T1 = 5.91	T2 = 2.72	T1 × T2 = 7.05	T1 = 5.86	T2 = 3.80	T1 × T2 = 7.96

*Refers to Se application without K-humate.

**Refers to the simultaneous application of Se and K-humate.

## Conclusions

This study focused on responses of oat plants to foliar application of Se (12 × 10^−3^, 63 × 10^−3^, and 88 × 10^−3^ mM) with and without the simultaneous application of K-humate (2.3 mM). Several parameters were used as relevant endpoints, including NPK concentrations in soil and plants, oat yield, soil organic matter, non-enzymatic antioxidants and total phenols, Se concentration in soil and plants, and Cr, Fe, Mn, Zn, and Cu in oat plant straw and seeds. Se supplementation increased the availability of N and P in soil and total concentrations in plant straw and seeds. The additional application of K-humate augmented these effects. Different concentrations of Se induced proportional increases in K-soil but not in plant straw or seeds. The application of K-humate with Se enhanced the effects in the soil but not in K-straw or K-seeds. The application of Se considerably improved the yield, but the simultaneous application of K-humate did not significantly augment this effect. Moreover, only significant responses were observed for seed productivity and plant length for Se application with and without K-humate. OMS was proportional to Se application with and without K-humate, as were total phenols. Conversely, the non-enzymatic antioxidant content was proportional to Se application, but K-humate addition showed no significant impact. The total Cr content was reduced by Se and K-humate application, and Fe in straw and seeds varied among treatments. Mn content of straw and seeds was reduced in response to Se and K-humate foliar application, and the middle concentrations of Se (Se2) produced the highest accumulation of Zn and the order of effects was in the following order Se2 > Se1 > control > Se3. Concentrations of Zn in oat seeds were reduced by Se supplementation. Increases in Se concentrations from 12 × 10^−3^ to 88 × 10^−3^ mM reduced Cu-seed, and Se application with K-humate produced only insignificant increases in the Cu-straw content in the following order: Se1 > Se2 > control > Se3. The additional application of K-humate altered this order to Se3 > Se1 > control > Se2.

Future investigations will be carried out to maximize the oat growth and productivity in marginal environments via foliar application of selenium and K-humate in which marginal water may be subject to be exploited as a result of global climate change.

## Data Availability

The datasets used and/or analysed during the current study available from the corresponding author on reasonable request.
